# Discovery of rare variants implicated in schizophrenia using next-generation sequencing

**DOI:** 10.20517/jtgg.2018.26

**Published:** 2019-01-20

**Authors:** Raina Rhoades, Fatimah Jackson, Shaolei Teng

**Affiliations:** Department of Biology, Howard University, Washington, DC 20059, USA

**Keywords:** Rare variant, schizophrenia, next-generation sequencing, rare variant association study, targeted resequencing, whole genome sequencing, whole exome sequencing

## Abstract

Schizophrenia is a highly heritable psychiatric disorder that affects 1% of the population. Genome-wide association studies have identified common variants in candidate genes associated with schizophrenia, but the genetics mechanisms of this disorder have not yet been elucidated. The discovery of rare genetic variants that contribute to schizophrenia symptoms promises to help explain the missing heritability of the disease. Next generation sequencing techniques are revolutionizing the field of psychiatric genetics. Various statistical approaches have been developed for rare variant association testing in case-control and family studies. Targeted resequencing, whole exome sequencing and whole genome sequencing combined with these computational tools are used for the discovery of rare genetic variations in schizophrenia. The findings provide useful information for characterizing the rare mutations and elucidating the genetic mechanisms by which the variants cause schizophrenia.

## INTRODUCTION

Schizophrenia (SCZ) is a serious psychiatric disorder that affects 1% of Americans^[1]^, and over 23 million cases are estimated in the world. The financial costs of the disease have been estimated to be $62.7 billion in the United States^[2]^. The economic burden of SCZ, including both direct costs and indirect costs, is estimated to be $155.7 billion dollars annually^[3]^. People with SCZ may experience hallucinations, delusions, disorganized speech and social withdrawal^[4]^. Psychiatric comorbidities in these patients include depression, anxiety and cognitive deficits^[5]^. The disease has great impact on the quality of life for the patients and their families. Approximately 40% of SCZ patients attempt suicide and are at eight-fold higher risk than the general population.

The underlying biological mechanisms of SCZ remain elusive. The disease is known to be accompanied by dysfunction in neurotransmission; morphological changes in prefrontal cortex, hippocampus, and striatum; and alterations in the signaling between brain regions as well^[6–9]^. Some environment factors such as childhood trauma can increase the risk for SCZ^[10]^. The twin and family studies suggest that the genetic heritability is the most significant risk factor for developing the disease. For example, monozygotic twins are more than twice as likely to be diagnosed with SCZ than dizygotic twins^[11]^. Determining the genetics of SCZ and complex disorders is further complicated by evolutionary genetic theory. Though it would seem that common complex heritable disorders, should be phased out through natural selection, several plausible theories have been used to explain this paradox, including balancing selection, heterozygote advantage, pleiotrophy, and polygenic models^[12]^. The polygenic model seems to be supported throughout the literature, however determining the precise number of loci that contribute to a particular complex disorders or traits has been challenging^[12]^. Many genetic approaches have been applied to the investigation of genetic variants that play an important role in the development of SCZ^[12]^. Genome-wide association studies (GWAS) have identified single nucleotide polymorphisms (SNPs) associated with increased susceptibility in SCZ^[13,14]^. The loci revealed by GWAS have diverse biological roles including neurotransmission, inflammatory response, glucose metabolism, and cell adhesion^[15]^. Several studies have demonstrated weak associations between common variants and major psychiatric disorders such as SCZ, bipolar disorder (BD) and major depressive disorder (MDD)^[16]^. However, most of the common variants have small or moderate effects on the disease risk, and a large proportion of the heritability remains unexplained.

Rare variants, usually defined as alleles with a minor allele frequency (MAF) < 1% in population, have been demonstrated to contribute to the missing heritability in SCZ. The use of next-generation sequencing (NGS) technologies like targeted resequencing, whole exome sequencing (WES) and whole genome sequencing (WGS) have allowed scientists to identify the rare variants and estimate the deleterious mutation load to investigate the contribution of rare mutation to the complex diseases^[17]^. In a SCZ study, WES for 2,536 cases and 2,543 controls showed enrichment of rare disruptive variants in genes related to calcium channels and the fragile X mental retardation protein^[18]^. Identifying rare variants is important for prioritizing potential pathogenic targets for diagnosis and drug discovery. This review will focus on the development of rare variant association tools and recent discoveries for investigating rare variants implicated in SCZ using NGS.

## COMMON VARIANTS *VS.* RARE VARIANTS

### Common *vs.* rare variant hypotheses for complex diseases

There are two contemporary hypotheses concerning the genetic contribution of sequence variants for common complex disorders such as cancer and SCZ^[19]^. The “Common Disease, Common Variant” (CD-CV) hypothesis argues that common diseases are associated with the common genetic variants. It means that a few variants with high frequencies in the population are the major contributors for a common disease with complex traits. However, the common variant can only account for a portion of heritability of many genetic disorders^[20]^. One investigation demonstrated that the association of common variants with many disorders and complex traits only accounted for approximately 30% of the heritability in SCZ^[21]^. This finding implies that the missing heritability is due to undiscovered common variants and rare disruptive variants. To address the missing heritability issue, the “Common Disease, Rare Variant” hypothesis suggests that multiple rare variants with relatively high penetrance contribute to the genetic component of common diseases. These genetic variations are usually found within less than one percent of the population. Some studies have demonstrated that rare variants could account for a substantial percentage of the missing heritability in complex diseases and phenotypes^[22]^. Determining the precise sources of missing heritability is a necessary hurdle to understand the etiology of neuropsychiatric disorders.

### GWAS and common variants

The CD-CV hypothesis provides the scientific paradigm for GWAS, which is a powerful tool for understanding the etiology of complex disorders^[23]^. GWAS usually applies SNP arrays to identify the target genes that may be involved with common diseases from the entire genome^[24]^. This methodology depends heavily on assessing the correlation of MAFs of different variants and determining whether they are correlated with a set of traits^[25]^. GWAS has elucidated many common variants in complex illnesses. For example, a GWAS study for Crohn’s disease identified over 30 candidate variants, and also demonstrated that variants in *ILK23R* and *IL12B* are also associated with psoriasis and other autoimmune disorders^[26]^.

GWAS has been used to identify many common variants associated with SCZ. Betcheva *et al.*^[27]^ performed a GWAS analysis to screen 554,496 SNPs in 188 SCZ patients and 376 controls from Bulgaria. One SNP, rs7527939, in the *HHAT* gene demonstrated a significant association with SCZ with an odds ratio of 2.63. Previous work has shown that the microstructure of white matter is altered in the brains of SCZ, specifically in the left and right anterior cingulate, left and right posterior cingulate, the inferior parietal cortices than was present in unaffected controls. Univariate association analysis showed that one variant upstream of the *CXCR7* gene, was associated with a reduction in white matter, and a multivariate analysis revealed an association with the *SORCS1* gene^[28]^, which lends support to the polygenic etiology of SCZ^[29]^. Many of the genes affected by these common variants are known to play a role in many important cellular functions, such as mitotic arrest, signal transduction, voltage dependent calcium receptors as well growth and differentiation cells^[30]^. GWAS have also been instrumental in elucidating pathways that are enriched with SNPs associated with SCZ. These pathways include serotonergic signaling, ubiquitin mediated proteolysis, hedgehog signaling, adipocytokine signaling, and renin secretion. It is interesting to note that the SNPs that were enriched in the aforementioned pathways were all found in regulatory regions^[31]^.

There are limitations for using GWAS to investigate the role of sequence variants in disease. GWAS studies are based around the concept of linkage disequilibrium (LD), whereby alleles within a particular locus are generally more closely related than the alleles that are located more distantly. The strength of the LD is dependent on the frequency at which alleles appear within the population. The greater the allelic frequency or the more common the variation is, the stronger the association or LD. Thus, many of the SNPs identified as being associated with a particular trait are not likely to be causal due to LD^[32]^. Increasing the sample size significantly, however, will result in the selection of several common variants of small effect. Meanwhile, rare variants will be masked by or undetected by GWAS because of low statistical power, caused by small number of cases, low allelic frequencies, low prevalence rates, *etc*.^33,34^. In addition, sample size has an important effect on the results of common variant analysis in GWAS studies, such that small sample sizes will often result in the identification of few variants with large effects^[35]^.

### NGS and rare variants

Rare variants are generally not detectable by GWAS because of their low frequencies which makes detecting them much more difficult than common variants. Multiple NGS technologies have been developed to identify the rare variants including single nucleotide variants (SNVs) and copy number variations (CNVs). Target resequencing takes advantage of using multiple probes or multiplexed PCR techniques to enrich specific regions of genes and is far less costly than using custom arrays^[36]^. WES uses targeted gene panels to sequence the coding regions, approximately 2% of the human genome. It provides a less expensive way to search for sequence variants throughout the genome^[37]^. Finally, WGS can be used to search for variants throughout the entire genome^[37]^. It also captures the non-coding regulatory regions^[38]^, which are important for gene expression. However, prior knowledge regarding the functional relevance of sequences found in non-coding regions is necessary in order to collapse or aggregate these rare variants in a meaningful way^[39]^.

Rare causal variants can be hard to identify precisely because they are so uncommon due to evolution placing negative selection pressure on alleles with deleterious effects on fitness^[40]^. It is worth noting that complex disorders like SCZ seem to have escaped this negative selection pressure, suggesting that these disorders are polygenic or arising from multiple rare genetic variations. Although experimental strategies can help make the identification of rare variants easier, ultimately the application of computational statistical methods is required. NGS results in poor signal to noise ratio when used for detecting rare variants. Therefore, it is important to use targeted enrichment methods such as targeted resequencing or WES, along with computational tools such as annotation databases and softwares and statistical methods^[38]^. Even when these methods are applied, the effect sizes of rare variants are expected to be moderate to weak when compared to common variants. So determining the sequencing depth is an important decision in terms of experimental design, as larger samples and fewer reads per base might be more desirable than high depth read of a few cases and controls^[40]^. Determining how one should annotate sequences is also critical to the analysis of rare variants, and many tools have been designed to identify different types of mutations and structural variations^[41]^.

## STATISTICAL TOOLS FOR RARE VARIANTS ASSOCIATION STUDIES

**Figure 1.**
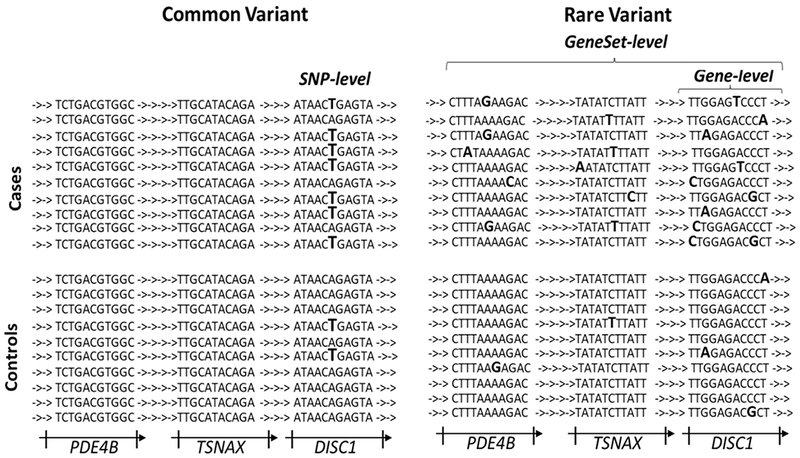
Statistical approaches for common and rare variant association studies. Common variant association studies can detect the association at the SNP-level. However, rare variant association analysis requires collapsing of variants into groups such as gene sets or genes. SNP: single nucleotide polymorphism

Different statistical approaches have been developed to investigate the associations between common and rare variants and genetic disorders [Figure 1]. The single marker testing is usually applied in GWAS to identify the common variants associated with complex disorders. In this approach, the genotypes of each SNP are coded and their effects are computed based on the number of variants in case and controls using the statistical tests such as Student’s *t*-test and Fisher’s exact test^[42]^. The rare variant association study (RVAS) methodologies require much larger samples sizes than GWAS because the signal is lower overall for rare variants than for common variants. Due to low statistical power, it is not possible to determine the effect size of single rare variants on complex diseases or phenotypes^[43]^. Therefore, the association approach was developed by grouping the rare variants across a genomic region, a gene or multiple genes^[40]^. Multiple marker testing evaluates the effects of multiple rare variants to determine whether they are associated with the disease or trait under investigation. The outcome of either test will depend greatly on whether the disease is the result of a single common variant or of multiple rare variants. Here we review the approaches that have been applied to the investigation of rare variants in genetic disorders in general, and how these tools have been employed for analyzing NGS data [Table 1].

### Approaches for case-control studies

RVAS approaches use statistical methods to combine the effects of rare variants to strengthen signals. The burden test is carried out by collapsing variants in a gene or functional region into a single score, then the association between the collapsed score and the phenotype is computed. The collapsing of the variants is accomplished either through the selection of a threshold or by the use of permutation tests, which require a lot of computational power^[41]^. Collapsing of variants into a single score results in each variant being treated as though it has the same effect on the phenotype. One way to test for the varying effects of rare variants on phenotype is to use the Multi-phenotype Analysis of Rare Variants (MARV) test. This is a type of burden analysis that utilizes multi-phenotype analysis. MARV calculates MAF at rare variants within a region of the genome. It then performs a linear regression to associate phenotype or combinations of phenotypes with the MAF for each variant^[44,45]^. The cohort allelic sums test is a burden test that collapses variants into a single score and can identify genes that carry one or more risk alleles. The score indicates the presence or absence of a minor allele, which is then tested for its association with a phenotype using univariate analysis^[46]^. Meanwhile, the combined multivariate and collapsing method combines the collapsing of rare variants with multivariate analysis of both collapsed rare variants and uncollapsed common variants^[47]^.

Note that the burden test assumes that all rare variants contribute the disease in the same direction. However, most rare variants have small effects on disease. In addition, some variants are disease-causing mutations, and some are protective variants. To address this scenario, the variance-component tests were developed. The C-alpha test can be used to determine the directionality of an effect of multiple variants on a phenotype. The generalized C-alpha test generates scores, based on summary statistics, which evaluates the increasing and or decreasing effects of multiple variants on a phenotype based on the Gaussian distribution. The phenotype can be binary or quantitative. The C-alpha test can be used on large population datasets or on smaller samples, such as familial studies. The C-alpha test operates on three basic assumptions: the number of variants, strength and independence of the effects, and the assumption that the variants are normally distributed^[48]^.

The Sequence Kernel Association Test (SKAT) is a type of supervised machine learning, that evaluates each variant based on a *P* value and then weights each variant based on a linear or logistical regression model^[49]^. One advantage of SKAT is that it allows for the detection of interactions between variants^[50]^. This method also allows for the epistatic effects to be revealed. However, in the case where many rare variations are in a particular region and have a similar effect on the phenotypes, the optimal unified test, called SKAT-Optimal Unified Test (SKAT-O), can be used. Rather than using collapsed scores, SKAT-O utilizes the minimum *P* values from different kernels, which include correlation effects^[50]^. The SKAT-O program, due to its power. When sample sizes are insufficient to generate accurate *P* values, the adaptive procedure-SKAT (AP-SKAT) can be used. This software is similar to SKAT, except that it “adaptively stops” the permutation test when the *P*-value is outside of the confidence interval of the *P* value that would be predicted using a binomial distribution. This procedure can be used to reduce the risk of obtaining a type I error^[51]^. AP-SKAT is more efficient in terms of computation than either SKAT or SKAT-O^[47]^. To investigate the dataset with fewer samples than required by SKAT, exact variance component tests can be used^[52]^. These tests minimize type 1 errors and in small samples, these types of tests identify more genes associated with polygenic traits that are identified by SKAT. Gene association with multiple traits is a variation of the sequence kernel association, called kernel distance covariance. This test uses non-parametric tests to test the association between rare variants and multiple phenotypes^[53]^. Similarity and dissimilarity are assessed for both genotype and phenotype and a matrix is formed for each variable. Then, the similarity or dissimilarity matrices for each variable are tested for independence. The calculation of *P*-values does not require any permutations and the method can be utilized on WES or WGS^[53]^.

### Tools for family studies

Family-based study designs are extremely advantageous to the study of rare variants because the frequency of rare alleles for a particular illness or disorder will be higher in a pedigree than among unrelated individuals^[54]^. Currently, there are very few rare variant analysis tools that are designed to find associations within sequences from family studies. The sampling of relatives in sequencing studies can help one to avoid sequencing errors in the analysis^[55]^. Therefore, the Minimum *P*-value Optimized Nuisance parameter Score Test Extended to Relatives (MONSTER) was developed. MONSTER is an extension of SKAT-O and tests for the association between rare variants and a phenotype, however, it can correlate data based on kinship^[55]^. It combines the SKAT model with a burden test model, where depending on the dataset presented ρ will either be equivalent to zero, as in family-based SKAT (famSKAT), or equal to 1 as is the case with family-based burden test (famBT). famSKAT is a statistical strategy that uses sequence kernel association to evaluate rare variants in samples that contain related individuals^[56]^. FamBT is a burden analysis that can be used to evaluate associations between rare variants and phenotypes when samples contain kin. However, MONSTER is capable of adaptively switching between models, performing like either famSKAT or famBT depending on the data imported^[55]^.

A particular challenge in conducting a rare variant analysis of pedigree sequencing data is identifying *de novo* mutations^[57]^. Pedigree Variant Annotation, Analysis, and Search Tool is one of the tools that exists for rare variant analysis of familial data, it uses both association testing and the logarithm of odds (lod) scores to identify rare causal variants from familial data^[57]^. Fampipe is a pipeline that can be used to analyze rare variant data from association studies, the pipeline can calculate identity by descent scores as well as lod scores to identify regions that demonstrate association^[54]^. The pipeline has several modules capable of calculating allelic frequency, family-specific mutations and more. To analyze binary traits in familial based studies, the Kernel Machine Generalized Estimating Equations model (GEE-KM) was developed^[58]^. The Rare Variant association analysis with Family data (RVFam) package for R analyzes SNP for associations with either continuous, binary, or survival phenotypes in familial sequencing studies^[59]^. The family-based association tests (FBAT)^[60]^ collapse variants using the sums of allele frequencies to generate test statistics that are weighted. These weighted summed stats are then tested for association with phenotypes using either multiple regression, linear regression, or linear combination analyses. Family-based Rare Variant Association Test^[61]^ is an extension of FBAT, a burden test with a variance component that can be used for rare variant association testing within extended families. The RVAS approaches can also be used to investigate rare and *de novo* noncoding variants in family studies. An analytical framework has been developed to investigate the *de novo* variations from WGS data in autism spectrum disorder (ASD) families^[62]^. The SNVs and indels are annotated and grouped by variant type, gene, species conservation, gene set, and regulatory region. The number of *de novo* mutations located in these regions in cases was compared to the number in sibling controls. Burden analyses are then performed to compute the significance of these comparisons. A similar procedure was used to detect the associations of *de novo* structural variants in different annotation groups. The authors analyzed rare variants in 519 ASD families and did not detect the significant association between rare *de novo* mutations in non-coding regions and ASD. However, they observed some biologically plausible associations that might warrant further investigation^[62]^.

## TARGET RESEQUENCING OF CANDIDATE GENES

Targeted resequencing was developed to sequence the target genes or regions of interest^[63]^. The primary advantage of the technology is that they allow for more targeted sequencing of specific portions of the genome. Targeted resequencing can be used to identify rare variants in candidate genes associated with SCZ [Table 2]. One strategy is to sequence a few extreme cases and then compare the rare variants identified in cases with a few controls. The other strategy is to use a few extreme cases to identify rare variants in novel genes and perform a sequencing on a large cohort, targeting the potential candidate gene^[64]^.

### Disrupted-in-Schizophrenia 1

Disrupted-in-Schizophrenia 1 (*DISC1*) is a candidate gene implicated in multiple major psychiatric disorders including SCZ, BD and MDD. It was originally reported in a linkage analysis of a translocation (1;11) (q42;q14) co-segregated with SCZ, BD, and MDD in a large Scottish pedigree^[65]^. Several additional independent studies have also reported associations between *DISC1* variants with other psychiatric disorders, endophenotypes, and neurophysiological traits^[66–68]^. Rare missense mutations in DISC1 have been identified in patients with SCZ, BD, and MDD. In a previous study, a targeted resequencing was used to sequence the DISC1 locus (528 Kb) in 1,542 samples^[69]^. Two-thousand and ten rare variants (MAF < 1%) were found, and 489 variants were located in regulatory regions while 36 mapped to coding exons^[69]^. One variant, rs16856199, in DISC1 was found to be significant in a region-wide association test and burden analysis revealed an excess of rare regulatory variants in MDD patients. In addition, a rare missense mutation R37W in an MDD patient was found to be transmitted to two affected offspring.

### DISC1 pathway

DISC1 functions as a scaffold protein in a large pathway comprised of two networks of genes, termed the “DISC1 Interactome” and “DISC1 Regulome”. The “Interactome” describes a set of proteins that interact directly with the DISC1 protein, whilst the “Regulome” designates a set of genes that are regulated by DISC1 protein. Both networks converge on pathways critical for various neurodevelopmental processes and therapeutic targets of of SCZ, BD, and MDD. Genetic analyses of patients with psychiatric disorders have identified additional mutations in DISC1 pathway genes. For example, the GWAS studies have found common variants in SCZ candidate genes like, *PDE4B*, and sequencing of *DISC1* and genes within the DISC1 pathway has revealed an excess of rare missense variants in SCZ cases versus controls^70,71^. Variations within the DISC1 pathway can have numerous effects on cognition as well as neurodevelopmental processes and signaling. Perturbations in the DISC1 pathway could represent an important susceptibility pathway in the development of SCZ.

In a recent study, a targeted resequencing analysis of 59 “DISC1 Interactome” and 154 “DISC1 Regulome” genes was performed in 654 cases with SCZ, BD or MDD, and in 889 healthy controls^[72]^. Burden analysis showed the gene translin-associated factor X-interacting protein 1 (*TSNAXIP1*) was enriched for rare damaging mutations in SCZ and MDD cases relative to controls. SKAT analysis showed an increased burden of singleton disruptive variants in the “DISC1 Regulome” in SCZ patients. There are significant associations between rare functional variants in the interactome with measures of cognitive performance. *DISC1, MAP1A, CIT*, and *DST* were found to be associated with cognitive ability based on burden analysis. Taken together these data suggest that genes in the “Regulome” and “Interactome” play a role in the etiology, progression, and severity of symptoms in SCZ^[72]^.

Targeted resequencing has been applied to sequence DISC1 and its interactors for SCZ and other neurodevelopmental disorders in other studies. Kenny *et al.*^[71]^ sequenced patients with ASD and SCZ in 215 synaptic genes including DISC1 Interactome genes known to be important to synaptic function. A significant burden of loss of function variants were found among combined cases of ASD and SCZ patients, which demonstrates that dysregulation at the synapse is an important feature of ASD and SCZ^[71]^. Moens *et al.*^70^ investigated rare coding variants in 10 DISC1 Interactome genes in 486 SCZ patients and 514 healthy controls from a Northern Swedish population. They found that rare mutations with MAF of 0.01 or less were found more often in SCZ cases than in controls. Unrelated studies of drug dependence have also demonstrated the significance of DISC1 pathway genes within the regulome as well, showing for instance, significant associations between rare variations found in *DISC1* and *GRIN2B* and opioid dependence^[73]^.

### Other candidate genes and pathways

Several other candidate genes have been implicated in SCZ, with a wide variety of functions. Rare variations in nuclear distribution E homolog 1 (*NDE1*), a gene that plays a role in microtubule formation, mitosis, and neuronal migration^[74]^. One investigation identified variant S214F, which was significantly associated with SCZ based on Fischer’s exact test. This variant is deleterious to the function of the NDE1 protein^[74]^. The targeted sequencing of Northern Swedish SCZ patients revealed that *NDE1* is significantly associated with SCZ diagnosis^[70]^. Dysfunction in myelination of axons, has also been implicated in SCZ patients. A rare variant in a receptor subunit for a myelin-associated inhibitor known as RTN46 was shown to have a modest association with SCZ^[75]^. This subunit has been demonstrated to play a role in synapse formation and is located on chromosome 22 in a mutational hotspot associated with SCZ. Upon further investigation, this variant was found to affect synaptogenesis by decreasing growth cone collapse^[75]^. Imputation analysis of GWAS data found both common and rare variants in *ADAMTSL3* gene, a constituent of the extracellular matrix^[76]^. An additional investigation found four rare variants located in noncoding regions near the *MIR185* gene, which codes for a microRNA with many functions^[77]^.

There is an overlap in many of the genes found to contain rare variation in SCZ with other neuropsychiatric and neurodevelopmental disorders like ASD. *GRIN2B* is a gene that is regulated by the DISC1 protein, implicating dysfunction in glutamateric signaling in SCZ neuropathology. In a study on ASD and SCZ, the investigators found an excess of rare variants, over 200 non-synonymous variants. Many of these rare variants had deleterious effects on genes associated with neurite outgrowth, Rett syndrome, and intellectual deficits^[78]^. Further investigations of the genetic overlap between SCZ and ASD have also demonstrated that the rate of *de novo* mutations in ASD spectrum disorder and SCZ patients was higher than that of controls^[79]^.

## WES FOR CODING VARIANTS

WES is a method of sequencing that focuses on coding regions of the genome. It is cheaper than WGS, making it possible to sequence more samples, while potentially increasing the signal to noise ratio. However, the power of a WES study will depend on the number of samples and the amount of variation in the genes of interest^[39]^. By excluding *de novo* mutations and filtering of these variants in GWAS or WES can help to further narrow down the search for rare causative variants or as a means of grouping variants for association analyses^[80]^. Here, we review the WES studies for investigating rare coding variants associated with SCZ [Table 2].

### Case-control WES studies

WES has been widely used in case-control studies to search for candidate genes associated SCZ. In a WES study of 4,264 SCZ cases and 9,343 controls, investigators found a significant association between loss of function mutations in *SETDIA* and SCZ. *SETDIA* is a gene that is associated with developmental disorders, which is further underscored by the fact that loss of function mutations in this gene are rare within the general population^[81]^. Damaging and disruptive ultra-rare mutations were found to be enriched in cases of SCZ. Investigators observed *de novo* nonsynonymous mutations in two genes previously identified as SCZ candidate genes, *TAF13* and *SETD1A*. Additionally, A single ultra-rare variant in *NRXN1* was identified to be associated with SCZ^82]^. Giacopuzzi *et al.*^[83]^ sequenced an Italian cohort of 180 persons diagnosed with SCZ and detected 45 brain-expressed genes with deleterious effects. Four variants in four candidate genes (*ANO2, FMN1, MEGF8* and *GAD1*) were determined to have rare or novel missense mutations which were not detected in control patients. The results indicate that high levels of homozygosity are associated with increased rare recessive variations in genes that convey a higher risk of SCZ^[83]^. WES studies of common and rare variants in SCZ risk loci can be used to elucidate psychiatric drug targets and improve treatment outcomes. One such investigation found 10 of 167 pharmacologically relevant gene sets were enriched for deleterious rare variants in SCZ patients. When the investigators examined the enrichment of common variants in pharmacologically relevant gene sets, they found that 35 of 167 gene sets were enriched among SCZ patients. Interestingly, they found that when analyzing both common and rare variations, two gene sets of pharmacologically relevant drugs were associated with greater risk of SCZ, drugs that are meant to treat amoebiasis and protozoal diseases, as well as antipsychotics. Further analysis revealed 4 target genes associated with treatment efficacy in SCZ patients^[84]^.

Rare variations in genes that are intolerant to protein truncation variants have also been associated with increased risk in several neuropsychiatric disorders including SCZ^[85]^. Several pathways involved in cell cycle arrest, heterocycle metabolic processes, and covalent chromatin modification, and histone modification are enriched for rare functional variants. The findings demonstrated a role for dysfunction or dysregulation of the cell cycle and histone modification in the pathology of SCZ^[86]^. Rare disruptive mutations associated with SCZ are likely to play a role as patients have higher rates of rare disruptive mutation than healthy controls. Further investigation of three sets of genes associated with the activity-regulated cytoskeleton-associated protein (ARC), postsynaptic density 95 protein, and calcium ion channels, revealed that the number rare disruptive variants among these gene sets were increased in SCZ. Many of the rare disruptive variants found were non-synonymous *de novo* mutations^[17]^. In a case-control investigation, the scientists found that mutational rates for the diagnosed patients were correlated with advanced parental age. However, 79% of all *de novo* mutations occurred on paternal chromosomes. Patient exomes showed an increased rate in non-synonymous *de novo* mutations in genes associated with SCZ and synaptic function. Upon further investigation, it was shown that the proteins resulting from these non-synonymous mutations exhibited greater number of protein-protein interactions with synaptic proteins than was expected. Genes involved in forming the N-methyl-D-aspartate receptor (NMDAR) and ARC complexes, which are involved in processes underlying synaptic plasticity, were particularly enriched in non-synonymous mutations^[87]^. Many of the variations associated with SCZ are found within genes that are intolerant of damaging mutations. In a study of 9,274 controls and 4,133 SCZ cases, the authors compared rates of copy number variants between cases and control^[88]^. Investigators also performed joint analysis to determine if there were associations between cases and controls with respect to combinations of SNVs, copy number variants, and *de novo* mutations. The authors determined that SCZ patients had a greater number of variations in genes that are intolerant of loss of function mutations. Burden analysis also demonstrated that loss of function mutations were associated with intellectual disability in SCZ patients. Variations in several genes implicated in ASD were found to overlap with those associated with SCZ. The supervised learning method, gradient boosted trees, was used to determine whether combinations of inherited and *de novo* variants could be used to predict the risk of SCZ in WES data from 2,545 patients with SCZ and 2,545 controls^[89]^. The investigators reported that the algorithm was 85% accurate in identifying patients diagnosed with SCZ. The results support the polygenic model of SCZ. The investigators further argue that the results support the “threshold hypothesis”, whereby SCZ is mainly a result of the accumulation of inherited mutations from an unaffected parent in susceptibility genes beyond a threshold.

### Family WES studies

Familial studies can be advantageous in studying highly heritable disorders where the loci associated with diseases have been identified^[90]^. This strategy is likely to increase the signal of rare causal variants which may otherwise be dampened by the presence of common variants in association studies or burden analyses^[40,90]^. A previous WES study of 53 family trios of the first episode sporadic patients diagnosed as SCZ showed that rare *de novo* variants were 10 times more likely contain non-synonymous mutations than those inherited from parents^[91]^. In another investigation of *de novo* mutations in 14 trios, the researchers found the ratio of nonsense to missense mutations was higher than expected based on previous studies^[92]^. They concluded that *de novo* mutations likely accounted for the missing heritability in SCZ. Family studies can also be used to help identify genetic variants associated with complex disease within the general population. Investigators found consensus in *de novo* variations in the *PTPRG, SLC39A13*, and *TGM5* genes between in 5 proband trios and 12 out of 48 unrelated cases of SCZ^[93]^. Family cohort studies can aid the understanding of the heritability of SCZ within families. In a WES study of an Indian family, the investigators found the variants in *TIMP2* and *PIWIL3* genes are segregated with family members who had been diagnosed with SCZ^[94]^. Pharmacological targets can also be elucidated through family cohort studies. In an investigation of an affected Indian family, WES revealed a rare heterozygous missense mutation in the *TAAR1* gene, present only in the affected family members. This mutation affects a disulfide bond critical for the G protein-coupled receptor and may have relevance to the discovery of pharmacological interventions for SCZ^[95]^. A separate investigation of two cases and one control in a family identified four rare variations in *UNC13B* were unique to affected individuals^[96]^. In a study of three families affected by SCZ, *PDCD11* was found to have recurrent variations and three variants were determined to be deleterious^[97]^. *ATP1A3* is associated with alternating hemiplegia of childhood and was previously demonstrated to be associated with ASD. Chaumette *et al.*^98^ used WES to determine whether rare variations in this gene are associated with risk of childhood-onset SCZ in an American cohort. They found three variants, two *de novo* and one a non-synonymous, in the *ATP1A3* gene and three rare variants in an interacting gene *FXYD1*. These genes were associated with a greater risk of SCZ. *ITGB4* gene is involved in cellular interactions and is highly expressed in neural stem cells and precursors. A gene-wise weighted burden test conducted using Bulgarian proband trios failed to find any gene that achieved exome wide significance, however, *ITGB4* was the most “highly ranked” based on its signed log *P*-value^[99]^.

Family WES studies can be used to identify pathways associated with a greater risk for developing SCZ. A study of a familial cohort combined WES and identity by descent model to map alleles associated with greater risk of SCZ^[100]^. Non-synonymous variants that were associated with greater risk for SCZ and were also enriched in pathways involved in the extracellular matrix and the development of neuronal projections. Although, future investigations might consider whether this variation might contribute to certain cognitive or behavioral symptoms^[96]^. Timms *et al.*^[101]^ investigated rare variants associated with the NMDA receptors in 5 families whose pedigrees demonstrate several persons affected by SCZ. They found rare variations in 21 genes, two of which were found in *GRM5*, a metabotropic glutamate receptor. Both variants affect the extracellular domain. Rare variants were also determined to occur in the *PPEF2* and *LRP1B* genes as well^[101]^.

## WGS FOR GENOMIC VARIANTS

WGS can lead to the identification of associations between genomic variants and SCZ [Table 2]. It can be used to identify small variants, including SNV and small insertions and deletions (INDELs), in coding and noncoding regions. Since the entire genome is sequenced, WGS is also a comprehensive tool for detecting large structural variants such as CNVs.

### Small variants

WGS has given investigators the ability to investigate the association between rare small variations and disease. One investigation found deleterious missense mutations in the *SHANK2* and *SMARCA1* genes which play a role in the post-synaptic density and neurogenesis^[102]^. An investigation of a large Chinese family with several members diagnosed with SCZ found that the genetic model of inheritance was autosomal dominant and one missense variant in *RELN*, a gene involved in neuronal migration, was significantly associated with SCZ^[103]^. The missense variant in *RELN* was found to co-segregate with the disorder and to be disruptive to protein function WGS of monozygotic twins discordant for SCZ indicates multiple genetic risk factors for SCZ^[104]^. The rate of *de novo* mutations was correlated with paternal age at the time of conception. A combination of inherited and *de novo* rare mutations seems to confer an increased risk of developing SCZ.

### Structural variants

The 22q11.2 deletion syndrome is a common genetic disorder, which is associated with a 25-fold increase in the risk of psychotic disorders, like SCZ^[105]^. In order to study the effects of 22q11.2 deletions on phenotype and develop better diagnostic assessments, the International Brain and Behavior Consortium developed a framework for investigations of the genetics of this disorder using data from patients and unaffected adults^[106]^. The model assumes that remaining haploid copies of segments of 22q11.2 deletion sites will demonstrate an increased number of variants in individuals affected by SCZ or psychotic disorders. The combined model also tests for SNPs, for and rare structural variants, which exist outside of deleted 22q11.2 regions. The findings showed that patients with SCZ had significantly more CNVs than undiagnosed individuals^[106]^. 22q11.2 deletions alone cannot explain the risk of developing SCZ. Investigators performed WGS in a group of nine unrelated patients, and demonstrated a greater burden for rare variants in cases than in controls in neurofunctional gene sets^[107]^. Burden analysis also revealed a large association between 22q11.2 microdeletions and rare variations in non-coding RNA genes as well. A familial study detected associations between variations in *COMT* and *PRODH* with cognitive impairments. Both the *COMT* and *PRODH* genes are located in the 22q11 deletion region^[105]^. Merico *et al.*^[107]^ performed WGS on 9 unrelated Canadian patients with SCZ to investigate the impact of 22q11.2 deletions on gene expression. The investigators identified rare variants in coding regions that were predicted to have damaging effects under a haploinsufficiency model. They found that the patients with SCZ had more deleterious rare variations in neuronal genes associated with neural projection as well as neuronal function. The investigators then investigated the association between SCZ and rare variants in genes that are affected by *DGCR8*, a gene involved in miRNA biogenesis. They found that patients with 22q11.2 deletions, where the function of *DGCR8* was affected, were at greater risk of developing SCZ. These results lend further credence to the miRNA hypothesis of SCZ^[107]^.

WGS was also applied to family studies to discover variants or modifications that could explain the variability of SCZ phenotypes. Khan *et al.*^[108]^ performed WGS to search for CNVs in 91 families with members diagnosed as schizophrenic. They found that there was an average of 9 rare CNVs per patient. Intronic deletions were found to be 3 times greater than exonic deletions. The transmission rates for rare exonic CNVs from unaffected parents to affected children were higher than for unaffected children^[108]^. A WGS study on 660 proband trios found rare CNVs in 14 loci that overlapped with SCZ risk genes in nine children seven offspring of persons who had attempted suicide. Forty-five of the patients that previously attempted suicide were found to have CNVs in 65 genes known to be involved in neural development^[109]^. Piluso *et al.*^[110]^ investigated *de novo* CNVs in cis-regulatory elements using NGS data from 46 family trios where at least one member was diagnosed with SCZ. They found *de novo* CNVs in genes associated with SCZ, intellectual disability, neuronal, migration, and neural development: *CNTNAP2, MAGI1, TSPAN7, CAV1, CAV2, MET*, and *ZIC1*. Two *de novo* mutations were found to affect the hs1043 and hs582 regulatory elements^[110]^.

## CHALLENGES

One challenge of investigating the genetics of SCZ is that many of the variants, which seem to confer susceptibility to SCZ or to have demonstrated an association with SCZ overlap with other neuropsychiatric illnesses including BD and MDD. In an investigation of over 19,000 cases of SCZ and BD, the investigators identified 219 SNPs that achieved genome-wide significance. These variants were found within 6 regions of the genome, two which were previously associated with SCZ, *MHC, MAD1L1*; two previously associated with BD, *TRANK1, IFI44L*, one gene that was based on previous work was associated with both diseases, *CACNA1C*; and a novel risk gene, *PIK3C2A*, also associated with both SCZ and BD. None of the 6 genes identified demonstrated a significant difference in terms of odds ratio between SCZ or BD, which further demonstrates the genetic overlap between the polygenic nature of these disorders^[111]^. The complex genetics of SCZ has also been demonstrated by the fact that it is difficult to distinguish between SCZ, schizoaffective, schizotypal, and non-affective psychotic disorders, which is sometimes referred to as the SCZ spectrum. In an investigation of the polygenic nature of SCZ spectrum disorders, the investigators calculated polygenetic risk scores for each subject based on the genome-wide significance of SNP in cases *vs.* controls. Individuals who lacked a psychiatric diagnosis had significantly lower scores than those of cases who were diagnosed on the spectrum^[112]^. SCZ and ASD also share a number of similar endophenotypes with respect to executive function, cognition, and sensory deficits^[113]^. Alterations in the morphology and neurochemistry of several neuroanatomical regions are common to both SCZ and ASD, including the prefrontal cortex, anterior cingulate, posterior cingulate, claustrum, and right parahippocampal gyrus among others^[114–116]^. In terms of genetic overlap, studies have found increased number of *de novo* splice site mutations and CNVs associated with both SCZ and ASD^[117,118]^. The common susceptibility genes associated with SCZ and ASD are post synaptic density associated genes like *DLG2, NRXN1*, and *BDNF*^[119–121]^. Factors relating to prenatal development represent another point similarity in the etiology of ASD and SCZ, particularly with respect to inflammation and neuroinflammation^[121,122]^. It is also worth noting that maternal infection and autoimmune disorders are associated with greater risk of developing ASD and SCZ^[14,123]^.

Another challenge is determining the role of environmental factors. The rate of the immune system’s exposure to bacteria is higher early in life and decreases with age. Impaired immune response in persons has been hypothesized to play a role in the development of SCZ^[113,124]^. However, most studies implicate maternal infection as a greater risk factor than postnatal immune insults^[14,125]^. Inferior social status is an environmental factor that may also play role in the increased risk of SCZ diagnoses^[108]^. These differences in environmental conditions between racial and ethnic minorities may result in neurobiological changes as well. In two Canadian investigations of clients at risk for psychosis and SCZ and healthy controls, researchers found that immigrants demonstrated increased dopamine release from the striatum relative to non-immigrants^[126,127]^. However, the mean genetic variability did not differ among the ethnic groups^[128]^. The racial or ethnic differences in the incidence of SCZ diagnosis are studies, which have shown that socioeconomic status and perceived honesty of the patient may influence diagnoses. In one investigation interviewers diagnosed 19% of white participants as schizophrenic, however study interviewers diagnosed nearly half of the black participants^[129]^. Socioeconomic factors, age, substance use, and reports of symptoms such as hallucinations differed between the two groups. However, investigators found that the client’s race still increased the likelihood of SCZ diagnosis three-fold^[129]^. A literature review concerning the black Caribbean populations in the UK found several possible explanations for the increased incidence of SCZ in these populations relative to white British persons^[130]^. The data suggests a genetic component as the risk of SCZ increases with the relatedness of an individual to persons affected by SCZ. The risk of developing SCZ increases the most for individuals with affected members of their immediate family, from 6%-13%. Other possible explanations include a predisposition for migration in schizophrenic individuals, misdiagnosis, drug use, and various socioeconomic factors. In a retrospective cohort study, investigators examined racial/ethnic disparities in SCZ diagnoses. The authors examined three classes of factors related to SCZ diagnoses, predisposing factors, demographic measures like age and ethnicity; enabling factors, such as insurance status and socioeconomic measures; and need factors, such as symptoms and substance use. The investigators found that the predisposing factors of male gender, Hispanic ethnicity, and African American race increased the likelihood of SCZ diagnosis^[131]^. A study with found that while polygenic risk scores were efficacious in distinguishing schizophrenic patients from healthy controls of European ancestry, that polygenic risk scores were less efficacious with African American participants^[132]^.

Detection of transcriptional signatures of SCZ provides new insight into the genetics basis of the diseases. To study the role of the microbiome in the etiology of SCZ, Olde Loohuis *et al.*^[133]^, collected the blood of 192 participants including patients with SCZ and BD and performed RNAseq to determine which microbes were present. Patients diagnosed with SCZ were found to have a more diverse microbiome than the other participant groups. After comparing SCZ cases to controls, the investigators determined that this was due to an overall increase in the microbial burden among SCZ patients^[133]^. A transcriptional analysis of lymphoblastoid cell lines from 268 cases and 446 controls showed 1,058 genes that were differentially expressed between SCZ cases and healthy controls^[134]^. A further analysis of the gene expression data found that SCZ cases demonstrated upregulation of genes related to immunological function and downregulation of genes related to apoptosis or non-immune functions^[134]^. Schizophrenics were also demonstrated to have a greater number of copies of the ribosomal RNA gene than healthy controls^[135]^. Brennand *et al.*^[136]^ used human induced pluripotent stem cells (hIPSC) derived neurons to model the neurobiology underlying SCZ. They found reduced arborization and synaptic contacts in the hIPSC neurons of SCZ patients, though spontaneous neuronal activity was found to be normal. They found 596 genes that were differentially expressed between the hIPSC neurons of SCZ patients and those of healthy controls. Treatment of hIPSC neurons with loxapine, an antipsychotic, increased the number of synaptic contacts and the expression of receptors crucial in glutamatergic signaling^[136]^.

## CONCLUSION

SCZ is a serious mental disorder affecting millions of people worldwide, but the underlying mechanisms remain unknown. The common variants identified by the GWAS only explain a small fraction of the estimated SCZ heritability. The development of NGS provides an unprecedented opportunity to identify the SCZ candidate genes and variants and research their functions. The analysis of the rare variants is timely and achievable in its aims of understanding the relationship between genotype and phenotype underlying SCZ. Multiple bioinformatics tools have been developed to detect rare variant associations in case-control and family studies. Targeted resequencing, WES and WGS have been used to identify the rare variants associated with SCZ. Although there are numerous challenges in SCZ research, the rare variant studies can provide useful information for characterizing the rare mutations and elucidating the genetic mechanisms by which the variations cause the SCZ and other mental illnesses. It could help biomedical scientists develop better diagnostic and treatments for individuals with psychiatric disorders.

## Figures and Tables

**Table 1.** Rare variants association tools

Tools	URL	Description	References
Burden test tools for case-control studies			
MARV	https://github.com/ImperialStatGen/MARV	Collapses rare variants into genomic region and performs analyses based on the proportion of minor alleles in rare variants	Kaakinen *et al.*^[45]^
CAST	https://rdrr.io/cran/AssotesteR/man/CAST.html	Variants are collapsed into a single variable and tested frequency association with the phenotype in question using univariate analysis	Morgenthaler and Thilly^[46]^
CMC	http://varianttools.sourceforge.net/Association/CMC	A method that combines collapsing with multivariate analysis	Li and Leal^[47]^
Variance component test tools for case-control studies			
C-Alpha	https://cran.r-project.org/web/packages/AssotesteR/AssotesteR.pdf	Analyzes the distribution of rare variants	Neale *et al.*^[48]^
SKAT	https://www.hsph.harvard.edu/skat/	Supervised machine learning test for the effect of multiple variants within a gene get or region	Wu *et al.*^[49]^
SKAT-O	http://www.hsph.harvard.edu/~xlin/software.html	Uses the values from different kernels, which include correlation effects	Lee *et al.*^[50]^
AP-SKAT	http://nagasakilab.csml.org/data/aSKAT.zip	“Adaptively stops” the permutation test when the *P*-value is outside the confidence interval predicted by the binomial distribution	Hasegawa *et al.*^[51]^
Exact VCTest	https://github.com/Tao-Hu/VarianceComponentTest.jl	Can be used with fewer samples than SKAT	Zhou *et al.*^[52]^
GAMuT	https://epstein-software.github.io/GAMuT/	Uses a non-parametric test to determine the association between rare variants and phenotypes	Chiu *et al.*^[53]^
Tools for family studies			
MONSTER	http://www.stat.uchicago.edu/~mcpeek/software/index.html	Extension of SKAT-O that can be used to correct for kinship	Jiang and Mcpeek^[55]^
famSKAT	https:///www.hsph.harvard.edu/han-chen/2014/07/31/famskat/	A statistical strategy that also uses sequence kernel association to evaluate rare variants in samples that contain related individuals	Chen *et al.*^[56]^
pVAAST	http://www.hufflab.org/software/pvaast/	Be used to evaluate associations between rare variants and phenotypes	Hu *et al.*^[57]^
GEE-KM	https://github.com/xfwang/	Analyzes binary traits in family studies	Wang *et al.*^[58*]*^
RVFAM	https://cran.r-project.org/web/packages/RVFam/index.html	SNP for associations with either continuous, binary, or survival phenotypes in familial sequencing studies	Chen and Yang^[59]^
FBAT	https://sites.google.com/view/fbat-web-page	A burden test with a variance component that can be used for rare variant association testing within extended families	Wang *et al.*^[60]^
FARVAT	http://healthstat.snu.ac.kr/software/farvat/	Collapses variants into weighted sum statistics that are tested for association using multivariate, regression, or linear combination analyses	Choi *et al.*^[61]^

MARV: Multi-phenotype Analysis of Rare Variants; CAST: cohort allelic sums test; CMC: combined multivariate and collapsing; SKAT: sequence kernel association test; Exact VCTest: exact variance component tests; GAMuT: gene association with multiple traits; MONSTER: Minimum *P*-value Optimized Nuisance parameter Score Test Extended to Relatives; pVAAST: Pedigree Variant Annotation, Analysis, and Search Tool; GEE-KM: Kernel Machine Generalized Estimating Equations model; RVFAM: R package for rare variant association analysis with family data; SNP: single nucleotide polymorphism; FBAT: family-based association tests; FARVAT: family-based rare variant association test

**Table 2.** Rare variants association studies

Design	Samples	Risk genes	Authors
Target resequencing			
Case-control	240 cases of SCZ, 221 cases of BD, and 192 cases of MDD and 889 controls	*DISC1*	Thomson *et al.*^[69]^
Case-control	80 SCZ cases and 80 controls	*DISC1, ATF5, GRB2, YWHAE, ZNF365*	Moens *et al.*^[70]^
Case-control	273 SCZ cases and 287 controls	*NRXN, NRIN, GRIN2B*	Kenny *et al.*^[71*]*^
Case-control	654 cases (241 SCZ, 221 BD and 192 MDD) and 889 controls	*DISC1, TSNAXIP1*	Teng *et al.*^[72]^
Case-control	760 cases with co-occurring alcohol dependence, cocaine dependence and opioid dependence, and 760 controls	*DISC1, GRIN2B*	Xie *et al.*^[73]^
Case-Control	433 SCZ, 145 pervasive developmental disorders, 3554 controls	*NDE1*	Kimura *et al.*^[74]^
Case-control	Set 1:370 SCZ, 192 ASD & Set 2: 1716 SCZ, 382 ASD, 4009 controls	*RTN4R*	Kimura *et al.*^[75]^
Case-control	92 SCZ patients	*ADAMTSL3*	Dow *et al.*^[76]^
Case-control	1000 SCZ cases and 500 controls. Follow-up analysis: 3598 and 4082 controls	*ATAT1, SH3PXD2A, NTRK3, MIR185*	Forstner *et al.*^[77]^
Case-control	142 ASD patients, 143 SCZ subjects, and 277 controls	*ILIRAPL1, MAOB*	Piton *et al.*^[78]^
Case-control	285 SCZ cases and 285 controls	*SHANK3, IL1RAPL1, NRXN1*	Awadalla *et al.*^[79]^
WES			
Mixed	4,264 SCZ cases, 9,343 controls and 1,077 proband trios	*SETD1A*	Singh *et al.*^[81]^
Case-control	12,332 individuals (4,877 SCZ patients, 6,242 controls and 1,144 other disorders)	*TAF13, SETD1A, NRXN1*	Genovese *et al.*^[82]^
Case-control	50 controls and 50 patients (from 180 SCZ patients)	*MEGF8, GAD1, FMN1, ANO2*	Giacopuzzi *et al.*^[83]^
Case-control	36,989 cases and 113,075 controls	*PSD-95, ARC, NMDAR*	Ruderfer *et al.*^[110]^
Case-control	100,296 individuals (mixture of cases and controls)		Ganna *et al.*^[85]^
Case-control	2,545 SCZ cases and 2,545 controls	*NSD1, HELLS, PHF21A, PAWR*	Curtis^[86]^
Case-control	2,536 patients and 2,543 controls	*ARC, FMRP, FMR1*	Purcell *et al.*^[14]^
Family	623 family trios	*ARC, FMRP, NMDAR* complex genes	Fromer *et al.*^[87]^
Meta analysis	Rare coding variants in 9,274 controls and 4,133 SCZ cases, de novo mutations in 1,077 family trios, and copy number variants from 6,882 cases and 11,255 controls		Singh *et al.*^[89]^
Case-control	2,545 cases and 2,545 controls	Selected genes implicated: *PLN, SLC25A4, NDP ARL1*	Trakadis *et al.*^[89]^
Family	53 family trios & 22 unrelated controls	Selected genes implicated: *ESAM, LAMA2, RB1CC1, SPATA*	Xu *et al.*^[91]^
Family	14 proband trios	Selected genes: *ZNF565, NRIP1, CCDC137, CHD4*	Girard *et al.*^[92]^
Mixed	14 parent child trios, 48 unrelated SCZ cases	*PTPRG, SLC39A13, and TGM5*	Kranz *et al.*^[93]^
Family	10 related individuals (3 diagnosed with SCZ)	*TIMP2*	John *et al.*^[94]^
Mixed	Multi-member family, 1000 cases & 1,050 ethnically matched controls, and 310 sporadic cases of Affrican American and Caucasian origin	*TAAR1*	John *et al.*^[95]^
Mixed	Multiplex family: 6 affected, 8 unaffected, 1 unknown, resequencing: 15 members of a multiplex family and 111 affected offspring	*UNC13B*	Egawa *et al.*^[96]^
Mixed	3 families with one affected patients and an unaffected sibling, 96 SCZ patients, 638 SCZ patients and 675 controls	*PDCD11*	Hoya *et al.*^[97]^
Mixed	Targeted sequencing of 2 cases of COS, WES 17 proband trios with sporadic COS	*ATP1A3 & FXYD*	Chaumette *et al.*^[98]^
Mixed	UK10K: 1,392 SCZ cases and 982 persons with severe childhood obesity; Sweedish SCZ Study: 2,545 SCZ cases and 2,545 control; Bulgarian trio sample: 591 SCZ trios, and UCL case control study: 1917 BD cases, 1304 SCZ cases and 1348 control	*IGTB4*	O’Brien *et al.*^[99]^
Mixed	161 affected patients and 69 controls	*FMRP, VGCCs*	Salvoro *et al.*^[100]^
Family	5 large families with multiple affected patients	*GRM5, PPEF2 & LRP1B*	Timms *et al.*^[101]^
WGS			
Family	5 families with 3 or more affected individuals	*SHANK2, SMARCA1*	Homann *et al.*^[102]^
Family	A large Chinese family with several affected members	*RELN2*	Zhou *et al.*^[103]^
Family	8 families of monozygotic twins who were discordant for SCZ	*TTN, GCN1L1, GAD1, PLXNA2, RELN2, FEX1*	Tang *et al.*^[104]^
Family	Two trios of unaffected patients with children with 22q11.2DS	*COMT & PRODH*	Chunget *et al.*^[105]^
Case-control	1,616 samples	*DGCR8*	Gur *et al.*^[106]^
	9 unrelated individuals with 22q11.2 deletions	*DGCR8*	Merico *et al.*^[107]^
Case-control	188 patient, 376 controls	*HHAT*	Betcheva *et al.*^[27]^
Family	91 SCZ families	Selected genes implicated: *ABCA13, CNTNAP2, SHANK2, SHANK3, HHAT*	Khan *et al.*^[108]^
Family	660 proband trios with at least one suicide attempt	*NRXN1 & VIPR2*	Sokolowski *et al.*^[109]^
Family	46 proband trios	*CNTNAP2, MAGI1, TSPAN7, CAV1, CAV2, MET, ZIC1*	Piluso *et al.*^[110]^

ASD: autism spectrum disorder; BD: bipolar disorder; MDD: major depressive disorder; SCZ: schizophrenia; COS: childhood-onset schizophrenia; WES: whole exome sequencing; WGS: whole genome sequencing
